# Chronic Non-Gonorrheal Infections of the Prostrate Gland

**Published:** 1910-09

**Authors:** Edgar G. Ballenger

**Affiliations:** Lecturer on Genito-Urinary Diseases, Atlanta School of Medicine, Atlanta, Ga.


### CHRONIC NON-GONORRHOEAL INFECTIONS OF THE PROSTRATE GLAND.

BY EDGAR G. BALLENGER, M. D.

*Lecturer on Genito-Urinary Diseases, Atlanta School of Medicine, Atlanta, Ga.*

For the sake of convenience and clearness this subject will be divided into infections which produce inflammatory changes and pus, and those which do not. Tubercular infections will not be considered in this paper.

The use of vaccine therapy in genito-urinary infections has led to more careful diagnosis as to the exact organisms present, and has shown that many patients have infections with germs other than gonorrhoea, which perpetuate the symptoms thought to be caused by gonococci. In reality, the gonococci may have long ceased to be present and the so-called "gleet" and chronic prostatitis may be caused by the staphylococcic or colon bacillus group of organisms, or by other pyogenic bacteria. I do not for a moment wish to give the impression that gonococci are not most treacherous and often difficult to demonstrate, nor to lead my hearers to believe that after an ordinary negative examination the chronic affection may be declared non-gonorrhoeal. A careful and prolonged study of chronic affections will show that many of them—perhaps most of the long continued ones without recrudescences—are kept up by the staphlococcus or the colon bacillus. Not a few acute conditions also are intensified by secondary infections with these organisms. While in many cases of simple urethritis and prostatitis, both acute and chronic, they are the primary infecting germs.

Nottshaft grew cultures from 120 prostates from 12 to 30 years after the last gonorrhoea and found mucrococci 119 times, bacilli 15 times and other bacteria 14 times. Many similar statistics could be presented.

Keyes says: "The gonociccus aline, unaided by any predetermining cause, may excite an acute cystitis, in such cystitis the urine is acid and this purely gonorrhoeal cystitis recovers or is replaced by a secondary mixed infection so that the gonococci, if still present, can no longer be found. Hence that striking clinical condition, acute gonorrhoeal cystitis, may be accepted as purely gonococcal in origin; while nearly all of the chronic manifestations in the bladder are due to secondary infection by those baccilli coli and pyogenic cocci that everywhere follow in the wake of the gonococcus to perpetuate the inflammation inaugurated by it."

The severity of the symptoms of these non-gonorrheal infections ranges from the very transient irritations to active inflammations. Occasionally buboes may appear to develop spontaneously and suppurate; the uethral and prostatic symptoms being so mild that one might easily doubt the source of the infection. Now and then patients develop epdidymitis from excessive sexual excitement plus the latent staphylococcic or colon bacillus infections. Periodic urinary irritation is a common symptom with frequent and perhaps painful urination. Not infrequently there is sexual weakness or hyperesthesia of the deep urethra. The more severe conditions present symptoms quite identical with gonococcic infections and need no special mention here, as I wish to devote more time to the obscure and often overlooked chronic and sub-chronic affections.

It is chiefly, too, with the second class of patients—those with urethral and prostatic infections *without inflammation,* that I wish to deal as the condition heretofore has been little understood. The subject has afforded a very fascinating study since beginning the use of the Reichert dark field illumination and the dahlia staining method—which I will describe later—in studying the secretion expressed from these infected prostates. The work, I think, has practically cleared up the mystery of the majority of the congested verumontanum sexual neuroses and hyperesthesias of the deep urethra and neck of the bladder and leads to a definite pathology for conditions that were formerly believed to be purely of functional origin. About 225 patients have been studied in regard to these infections and a comparatively uniform symptom-complex has been found to exist in the majority of cases. Absolutely normal prostatic secretion contains no germs. Prostates acutely inflamed contain comparatively few germs. Prostates with these sub-chronic infections—as I have chosen to call them—contain almost no pus cells but myriads of germs. One hundred and ten patients have been found with this sub-chronic type of infection. The origin of these infections is usually one or a combination of the following: Gonorrhoea, simple urethritis, urethral instrumentation, prolonged ungratified sexual excitement or sexual excess, masturbation, rectal or anal lesions, such as hemorrhoids, fissues, ani, etc.

A chronically congested prostate, whatever the cause, seems to favor the growth of germs that lodge in it; whether these germs are of descending origin being eliminated through the kidney, as we know colon bacilli often are, or whether they pass directly through the mucosa in some not well-understood manner, as certain observers believe is possible, I am unable to say. In a few patients there was no cause to which might reasonably assign the infection. With so many possible sonrces, however, one often hesitates to express an opinion as to which is the actual cause.

The symptoms are usually those of a very mild urethritis with a little irritation at the neck of the bladder and mild symptoms sometimes referred from this region. A very common complaint is itching in the urethra, not severe as a rule, but often returning without any apparent reason. The patients also frequently complained of a sensation as if discharge were escaping from the urethra and yet, upon examination, no discharge would be found. Occasionally sharp fleeting pains were observed in the urethra, penis or prostrate.

A "morning drop" of mucus or muco pus was found in a considerable number of the patients, while in others it was persistently absent.

Well-marked sexual impotence was found in 11 out of 110 patients; about 76 experienced premature ejaculation and a few complained of pain or burning in the deep urethra during or immediately after orgasm. Mild rheumatism involving the knee, hip, shoulder, wrist, ankle and other joints was observed in 25

patients. Its chief characteristic was its mildness and persistency, being amenable to no ordinary form of anti-rheumatic treatment. Slight pain or aching in the lower part of the back was of frequent occurrence and was of the character often seen in chronic prostatitis.

The conditions I have previously considered sexual neuroses and hyperesthesia of the deep urethra, the disturbance being all out of proportion to the inflammatory products, have in every instance shown the prostate to be infected with these mild organisms.

In about 85 of the patients the urine contained a few mucus shreds; in about 22 it was microscopically clear, but at times it would become slightly cloudy or opalescent about like urine in which there is a slight precipitation or phospates. This cloudiness was due to micro-organisms which at times caused the urine to have an unusually disagreeable odor especially if it was allowed to stand for any length of time. A number of observers have found that bacteriuria due to colon bacilli produces an acid urine, but when due to staphylococci it is likely to be alkaline. This, as a rule, has been confirmed but exceptions are not unusual..

Time forbids more than a few statements regarding the bacteria found and the size and character of the prostates. Suffice it to say, in regard to the infections, that from microscopic studies of all of them and from culture of 36 grown by Dr. Edgar Paulin, cocci and colon bacillus groups which had for some reason become attenuated or the host had become partially immunized. Probably the former view is correct. The stephylococcus aureus, albus and the dominating organisms were those belonging to the staphylocitreus, and colon bacilli in several forms have been present singly or in various combinations but these combinations apparently have not affected materially the symptoms or course of the affections.

The prostates have nearly always been increased in size in direct proportion to the duration of the infection, where a definite origin could be ascertained. It is my opinion from a considerable study of this subject, which appeared in detail in *The New*

*York Medical Record,* that we have in these low-grade mildly irritating infections the true cause of the chronic progressive obstructive hypertrophy which occurs in elderly men. I regret that I cannot here present the evidence from which I have drawn this deduction. As the remainder of the time is short, I will hasten to describe the technic of staining the prostatic secretion.

An irrigation of the urethra and bladder is given according to the Janet-Valentine method with a normal saline solution until one to two quarts of the solution has been used. Having the bladder partly filled with the irrigating fluid, the prostate is then massaged and a drop of the secretion which appears at the meatus is placed on a slide; a drop of a freshly prepared, a 1 per cent. aqueous solution of dahlia is mixed with this drop of secretion. The rim of the cover glass placed over it is sealed in position by applying melted white wax around its rim with a camel's hair brush. As a confusing precipitate forms, at times and especially when the specimen contains urine, it should be placed under the lens of the microscope and allowed to remain in this position for 20 to 30 minutes or longer, to allow the precipitate to settle to the bottom and become quiet, otherwise the Brownian movement of these minute bodies might be mistaken for motile organisms. The germs remain motile for a few days to a week or longer and a positive diagnosis may be easily made by a series of subsequent examinations, when their motility differentiates them from the debris which settles and becomes motionless. If preferred the specimen may be viewed with the dark field illumination and the diagnosis thus made. Fixed smears may also be made and stained in the usual manner, but these rarely give as accurate an idea of the presence and number of micro-organisms as does the above methods.

